# Characterization of the *CCT* family and analysis of gene expression in *Aegilops tauschii*

**DOI:** 10.1371/journal.pone.0189333

**Published:** 2017-12-08

**Authors:** Xingwei Zheng, Xiaohua Li, Chuan Ge, Jianzhong Chang, Mengmeng Shi, Jianli Chen, Linyi Qiao, Zhijian Chang, Jun Zheng, Jiancheng Zhang

**Affiliations:** 1 Institute of Wheat Research, Shanxi Academy of Agricultural Sciences, Linfen, China; 2 Institute of Crop Science, Shanxi Academy of Agricultural Sciences/Shanxi Key Laboratory of Crop Genetics and Molecular Improvement, Taiyuan, China; 3 Dept. of Plant, Soil, and Entomological Sciences, University of Idaho, Idaho, United States of America; Institute of Genetics and Developmental Biology Chinese Academy of Sciences, CHINA

## Abstract

Flowering is crucial for reproductive success in flowering plant. The CCT domain-containing genes widely participate in the regulation of flowering process in various plant species. So far, the CCT family in common wheat is largely unknown. Here, we characterized the structure, organization, molecular evolution and expression of the CCT genes in *Aegilops tauschii*, which is the D genome donor of hexaploid wheat. Twenty-six *CCT* genes (*AetCCT*) were identified from the full genome of *A*. *tauschii* and these genes were distributed on all 7 chromosomes. Phylogenetic analysis classified these *AetCCT* genes into 10 subgroups. Thirteen AetCCT members in group A, C, H and G achieved rapid evolution based on evolutionary rate analysis. The *AetCCT* genes respond to different exogenous hormones and abiotic treatments, the expression of *AetCCT4*, *7*, *8*, *11*, *12*, *16*, *17*, *19*, *21* and *22* showed a significant 24 h rhythm. This study may provide a reference for common wheat's evolution, domestication and evolvement rules, and also help us to understand the ecological adaptability of *A*. *tauschii*.

## Introduction

Flowering is an important development event in plant life cycle, which guarantees the adaptation to special geographical environments and reproductive success. The first cloned plant flowering control gene is *CO* in Arabidopsis (*CONSTANS*). The C terminus of CO protein contains a motif including 43–45 amino acid residues [1]. Several genes regulating flowering contain this conserved motif, such as *CO*, *COL* (*CO—LIKE*) and *TOC1* (*Timing of* CAB *expression 1*). Hereafter, the genes with this structure domain are called '*CCT* genes' [2, 3]. *CCT* family genes can be divided into four categories based on the latest sequencing information of Arabidopsis, rice and barley and other species: COL (CONSTANS-like), PRR (Pseudo response regulators), CMF (CCT Motif) and ZCCT family [1–3]. In Arabidopsis, most of the *CCT* family genes including *COL1*, *COL2* and *COL3* that can react with those downstream genes as *FT* (Flower time) and *SOC1* (Suppressor of overexpression of *CO1*) under the control of *COP1* (Constituitive photomorphogenic), affecting the plant flowering process under different light conditions [3]. CCT family members also participate in the heading stage of rice. *Hd1* (Heading date 1) is the first cloned *CCT* gene regulating flowering in rice [4]; in addition to affecting the height and ear length of rice, *Ghd7* also regulates flowering under the action of the ELF (Early flowering) family gene [5]. The *OsCO3* modulates photoperiodic flowering in rice, affecting *Hd1* through dose effect [6]; *OsCOL4* increases the expression of *Hd1* and delays the heading stage under long-day and short-day conditions [7]. Recently, two domesticated members of the *CCT* family of genes (*DTH2* and *Ehd4*) have been cloned, and they regulate the heading stage under different light conditions through MADS-box transcription factors, to meet the regional growth adaptability of rice [8, 9]. Overall, the *CCT* genes are conserved and regulate flowering time in different species, but the specific functions of different members have a great variation across species and populations.

Common wheat is heterologous hexaploid, including A, B and D genomes. About 8,000 years ago, hexaploid wheat was produced through the hybridization of wild emmer and *A*. *tauschii* (DD). The addition of the D genome has greatly improved the adaptability and quality of wheat, promoting wheat to become the food crop with the widest planting area in the world [10]. In addition, there are fierce competitions for light, fertilizer, water and other resources between *A*. *tauschii* and wheat, which decrease wheat production and even cause total crop failure. Due to there is no mature and effective control measure in production, *A*. *tauschii* becomes a weed during the wheat production process [11]. It occurs in the cornfields of many countries, and the damage degree is becoming more serious, with a trend of rapid spreading [12]. As such, an in-depth study into the adaptability and genetic evolution of the light cycle of *A*. *tauschii* can effectively control the harm, block the transmission and limit its spread and dissemination. At present, the composition of CCT family in Arabidopsis, rice and barley is basically clear, while a systematic analysis on the classification, quantity and function of the *CCT* genes in *A*. *tauschii* is still lacking. This article isolates *CCT* family genes (*AetCCTs*) and analyzes the chromosome location, sequence characteristics, gene structure, evolutionary rate, rhythmic expression and response to exogenous hormones using the bioinformatics method on the basis of the genome sequencing data of *A*. *tauschii*. The relevant results may provide valuable information for the further research and utilization of the *CCT* gene families of cereal crops, helping to understand the diffusion mechanism and spread of *A*. *tauschii* by weed type.

## Materials and methods

### Separation, structural analysis and chromosomal localization of the CCT sequences

The genome and protein sequences of *A*. *tauschii* were downloaded from the AGDB database (http://dd.agrinome./org), and a local protein database was established. The hidden Markov model file of the CCT family (PF06203) was downloaded from the Pfam database (http://pfam.sanger.ac.uk/), and the local protein sequence database was retrieved from HMMER software, and redundant sequences were removed. The obtained sequences were confirmed based on the presence of conservative domain of CCT proteins using the SMART database with an E-value cutoff of 1.0 (http://smart.embl-hei-delberg.de).

The sequence fragment location of *A*. *tauschii* and wheat EST markers information were sourced from the graingene 2.0 database (http://wheat.pw.usda.gov/). The gene structure was analyzed with GSDS software (http://gsds.cbi.pku.edu.cn/). The *CCT* gene sequences were submitted to the graingene 2.0 database, retrieving the *A*. *tauschii* sequences with a similarity of > 95% and E values of 0.0; the related EST sequences were obtained via genome sequence alignment. The *CCT* genes were named according to their chromosome location, and members of *AetCCT* were integrated into the related molecular map according to the location information of the sequence fragment and the information of the EST marker.

### Physical and chemical property analysis, sequence analysis and phylogenetic analysis of proteins

The relative molecular weights, isoelectric points and other information of the CCT protein sequences were obtained from the Editseq software of DNAstar. The conserved motifs among CCT members were identified using the MEME tool (http://meme.nbcr.net/). The parameters were set as follows: width of each motif was 10–300 amino acid residues, maximum number of motifs was 4, and other parameters with default values.

The sequences of Arabidopsis, rice and wheat CCT proteins were downloaded from NCBI (http://www.ncbi.nlm.nih.gov/). A total of 98 CCT proteins of related plants were selected for the phylogenetic construction, including 26 from *A*. *tauschii*, 22 from Urartu, 30 from rice and 23 from Arabidopsis. Multiple sequence alignments of CCT protein sequences were performed using ClustalX software, and a phylogenetic tree was constructed using the Neighbor-joining Method. The phylogenetic analysis were analyzed using MEGA software with the bootstrap value set to 1,000. The evolution rates (branch models) of each group in the phylogenetic tree were assessed under the tree branch model using PAML software. The positive selection sites were detected with the Branch-site Model.

### Plant materials and treatments

*A*. *tauschii* Y2282 seeds were sterilized with 75% alcohol and 10% sodium hypochlorite, rinsed for 5 times with distilled water, and placed on moistened filter paper in Petri dishes and cultivated in a growth chamber with 16 h light, 8 h dark photoperiod at 25°C. Following 10 days of growth, seedlings were immersed in 250 mM NaCl and 15% PEG 6000 for 72 h as salt and drought treatments, respectively. Plants were subjected to sterile water for control. Treated and control seedlings were harvested at 24 h and 72 h after treatment.

Responsive expression to hormone: plants of *A*. *tauschii* Y2282 (AL8/78) at the booting stage were treated with exogenous hormones, and the following solutions of each hormone were sprayed onto three replicate whole plants: Auxin (IAA, 2 mmol·L^-1^), Brassinolide (BL, 1 mmol·L^-1^), Gibberellic Acid-4 (GA4, 1 mmol·L^-1^), Naphthylacetic Acid (NAA, 5 mmol·L^-1^), Methyl Jasmonate (meja, 1mmol·L^-1^) and Salicylic Acid (SA, 15 mmol·L^-1^). Distilled water was served as the control. Roots, stems, flag leaves and young panicles were harvested separately at 24h and 72h after treatment.

Rhythm expression: *A*. *tauschii* Y2282 was vernalized at 4°C after sprouting, treated with a long photoperiod of daylight for 16 hours per day. Roots, stems, flag leaves and young panicles were collected from 3 individual plants at the jointing stage once every 3 hours, which lasted for 48 hours.

All samples were immediately immersed in liquid nitrogen and stored at -80°C until RNA isolution. All experiments were repeated 3 times.

### Total RNA extraction and real-time fluorescence quantitative analysis of gene expression

Total RNA was isolated with RNAprep pure plant kit (Tiangen) and quantified spectrophotometrically. cDNA was synthesized with M-MLV Reverse Transcriptase (Promega), the cDNA was diluted 10 times. Quantitative real-time PCR was performed according to the Takara SYBR Premix EX Taq instructions on a 7300 Real-time PCR System (Applied Biosystems). The glyceraldehyde-3-phosphate dehydrogenase (GAPDH) gene was used as an internal control, and the relative expression of the target gene was calculated according to the 2 –^ΔΔCT^ method. Primers used in qRT-PCR were listed in S1 Table.

## Results

### Identification and chromosomal location of *AetCCT* genes

Using the HMM searches and domain confirmation, we identified 26 putative CCT protein sequences in *A*. *tauschii* and named *AetCCT1* to *AetCCT26*, based on the order of their chromosome locations in graingene 2.0 database. The protein molecular weight of AetCCT ranges from 14.9 kD (AetCCT3) to 83.2 kD (AetCCT12), and the values of isoelectric point ranges from 4.2 (AetCCT13) to 10.2 (AetCCT14) (Table 1). The corresponding CDS and genomic sequences of *AetCCTs* were also downloaded.

**Table 1:** Characteristics of *CCT* gene family members in *A*. *tauschii*.

Gene	CDS	Scaffold	Chromosome	Protein length (aa)	Molecular weight (D)	Theoretical *Pi*
*AetCCT1*	AEGTA18365	scaffold4528	1DL	394	41364	4.27
*AetCCT2*	AEGTA21580	scaffold71509	1DL	289	30459	7.67
*AetCCT3*	AEGTA27323	scaffold97182	1DL	130	14871	9.66
*AetCCT4*	AEGTA10532	scaffold38896	2DS	625	67304	8.4
*AetCCT5*	AEGTA13911	scaffold30755	2DS	311	32906	7.44
*AetCCT6*	AEGTA00203	scaffold30755	2DS	224	24085	8.17
*AetCCT7*	AEGTA31460	scaffold77907	3DS	520	57947	6.34
*AetCCT8*	AEGTA06337	scaffold4745	3DL	307	32767	6.23
*AetCCT9*	AEGTA28548	scaffold28581	4DS	623	68607	8.11
*AetCCT10*	AEGTA01709	scaffold50727	4DS	163	18201	6.38
*AetCCT11*	AEGTA21446	scaffold108	4DL	579	63545	6.05
*AetCCT12*	AEGTA31746	scaffold2864	4DL	763	83206	6.4
*AetCCT13*	AEGTA18304	scaffold98624	4DL	382	41056	4.2
*AetCCT14*	AEGTA13770	scaffold12030	4DL	194	22793	10.2
*AetCCT15*	AEGTA33063	scaffold24714	5DL	170	19459	9.96
*AetCCT16*	AEGTA03508	scaffold185863	5DL	195	21577	7.94
*AetCCT17*	AEGTA04421	scaffold53469	5DL	649	71239	6.48
*AetCCT18*	AEGTA32221	scaffold7166	5DL	167	18809	8.71
*AetCCT19*	AEGTA05461	scaffold106936	6DS	482	52818	4.69
*AetCCT20*	AEGTA21198	scaffold137524	6DL	340	36722	4.96
*AetCCT21*	AEGTA15475	scaffold71269	7DS	392	43225	6.64
*AetCCT22*	AEGTA31079	scaffold66553	7DS	396	43609	4.81
*AetCCT23*	AEGTA08066	scaffold6305	7DS	292	31645	7.75
*AetCCT24*	AEGTA13638	scaffold7100	7DL	290	30683	9.55
*AetCCT25*	AEGTA03492	scaffold116052	7DL	228	23499	6.73
*AetCCT26*	AEGTA22574	scaffold16211	7DL	156	17219	9.22

**Fig 1:**
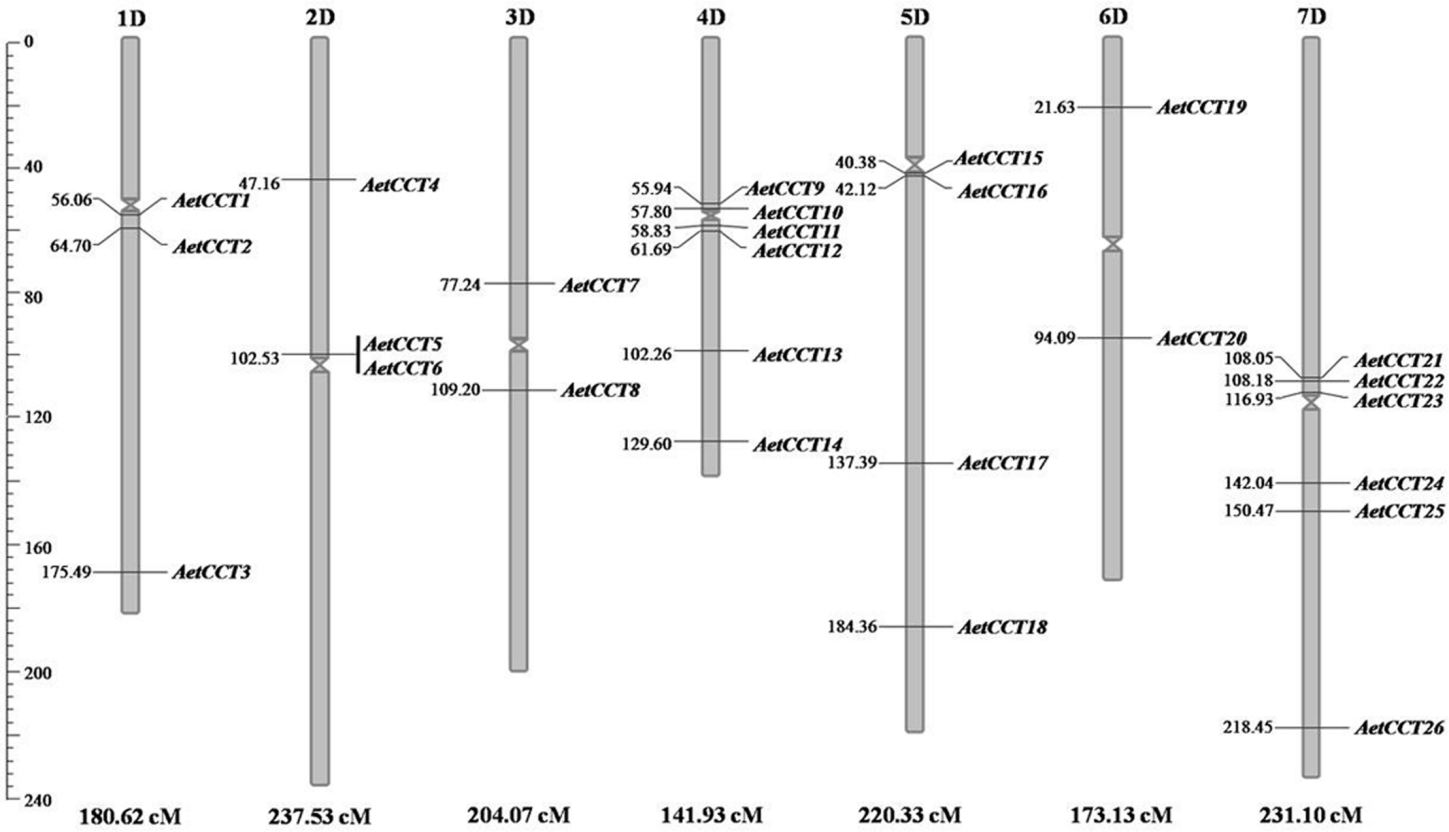
Chromosome distribution of *CCT* family in *A*. *tauschii*. The chromosome numbers are indicated at the top of each bar. The genetic position of each gene can be estimated using the left scale.

The 26 *AetCCT* genes are distributed among the 7 *A*. *tauschii* chromosomes (Fig 1). Chromosomes 4D and 7D contain relatively more *AetCCT* genes, with 6 members on each chromosome. The *CCT* gene numbers on chromosomes 1D, 2D, 3D, 5D and 6D are 3, 3, 2, 4 and 2, respectively. *AetCCT5* and *AetCCT6* are located on the same scaffold, as a pair of tandem repeat genes. In addition, 13 *AetCCT* members, for instance, *AetCCT1-2* on 1D chromosome, *AetCCT5-6* on 2D chromosome, *AetCCT9-12* on 4D chromosome, *AetCCT15-16* on 5D chromosome and *AetCCT21-23* on 7D chromosome are clustered in the region near the centromere.

### Phylogenetic analysis

**Fig 2:**
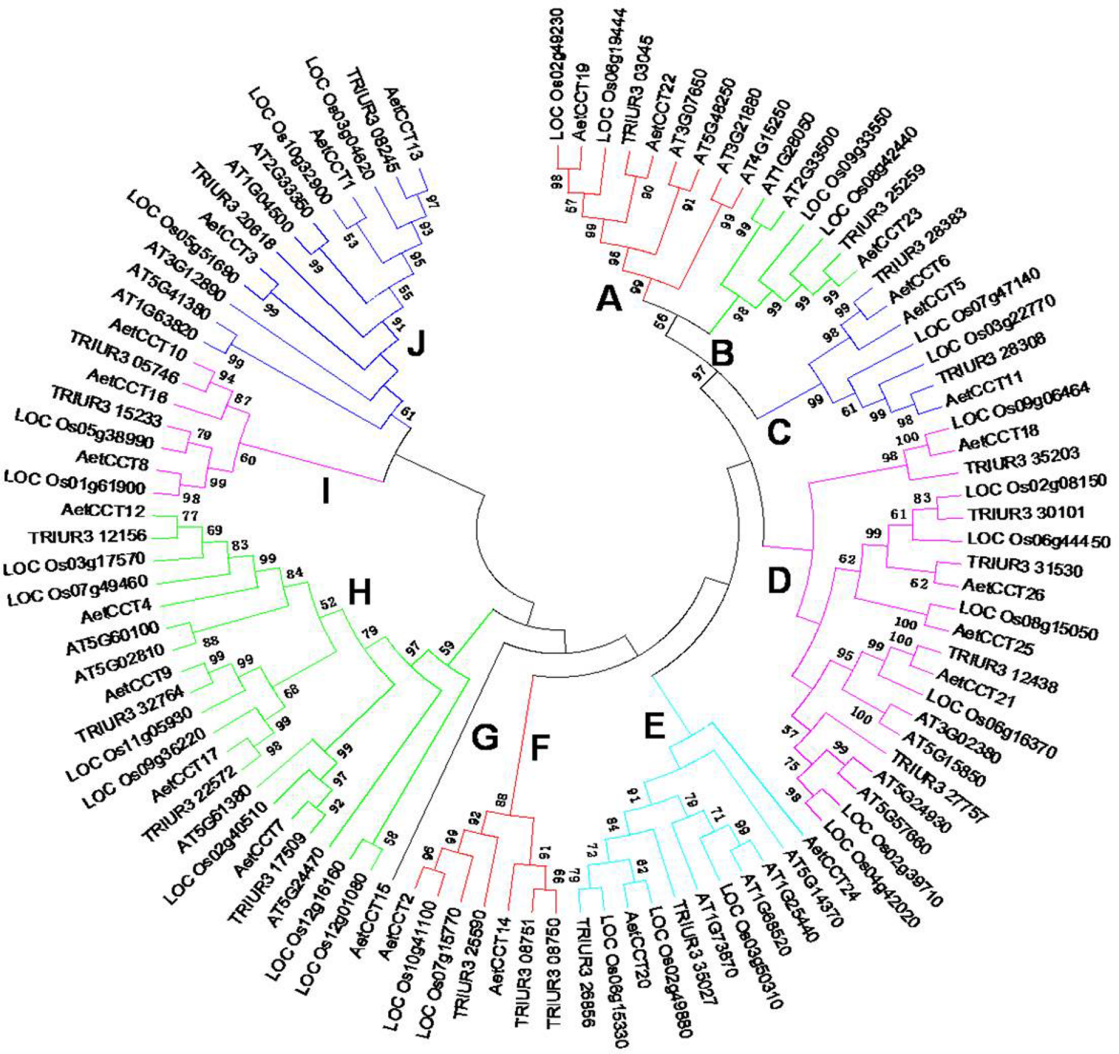
Phylogenetic relationship of CCT proteins among *A*. *tauschii* and other species. The full-length CCT amino-acid sequences of *A*. *tauschii*, *T*. *urartu*, Arabidopsis and rice were aligned by ClustalW and the phylogenetic tree was constructed using MEGA 6.0 by the neighbor-joining method with 1000 bootstrap replicates. The ten subgroups, Group A-J, are indicated with different colors.

A phylogenetic tree was established based on the alignment of the amino acid sequences of 101 *CCT* genes, including 26 from *A*. *tauschii*, 23 from Arabidopsis, 30 from rice and 22 from *T*. *urartu*. The results showed that the CCT family can be divided into 10 groups (from A to J, Fig 2). Of these *CCT* genes, groups A, C and D were identified as COL family, groups B, E, F, G, I and J were identified as CMF family, members of group H were classified as PRR family. The AetCCT family members were distributed in each branch of the evolutionary tree. Among them, group H have the largest number of AetCCTs, with 5 members (AetCCT4, AetCCT7, AetCCT9, AetCCT12 and AetCCT17), whereas group B and G have only one member in each group. AetCCT is the closest relative of *T*. *urartu* CCT, followed by rice and Arabidopsis. Six subgroups include Gramineae CCTs and dicotyledonous plants Arabidopsis CCTs, indicating that *CCT* gene amplification occurs before the monocot/dicot divergence.

Homologous genes with similar functions between species are usually clustered into the same group. For example, *AetCCT19* and *22* in group A may have similar functions as those of *AT3G07650* and *Os02g49230* (*OsDTH2*) in the same branch, making plants delay flowering under long-day conditions [9, 13, 14]. *AetCCT18*, *21*, *25* and *26* in group D may regulate flowering under short-day conditions like *Os09g06464* (*OsCO3*) and *Os06g16370* (*Hd1*) [15, 16]. *AT5G02810*, *Os02g40510*, *Os03g17570* and *Os07g49460* in group H are known as photoperiod control genes [17–19], and *AetCCT4*, *7*, *12* and *17* may also have a similar function, while they share an obvious circadian rhythm. In addition, it has been proven that *Os07g15770* (*GHD7*) in group F, *Os01g61900* in group I and *Os05g51690* (*NRR*) in group J can regulate rice flowering [5, 19], suggesting that AetCCT members in the same branch may participate in a similar flowering regulation pathway. The corresponding homologs in rice and Arabidopsis were listed in S2 Table [13, 15, 16, 20–24], the genes which have been investigated were marked.

### Gene structure and conserved motifs

**Fig 3:**
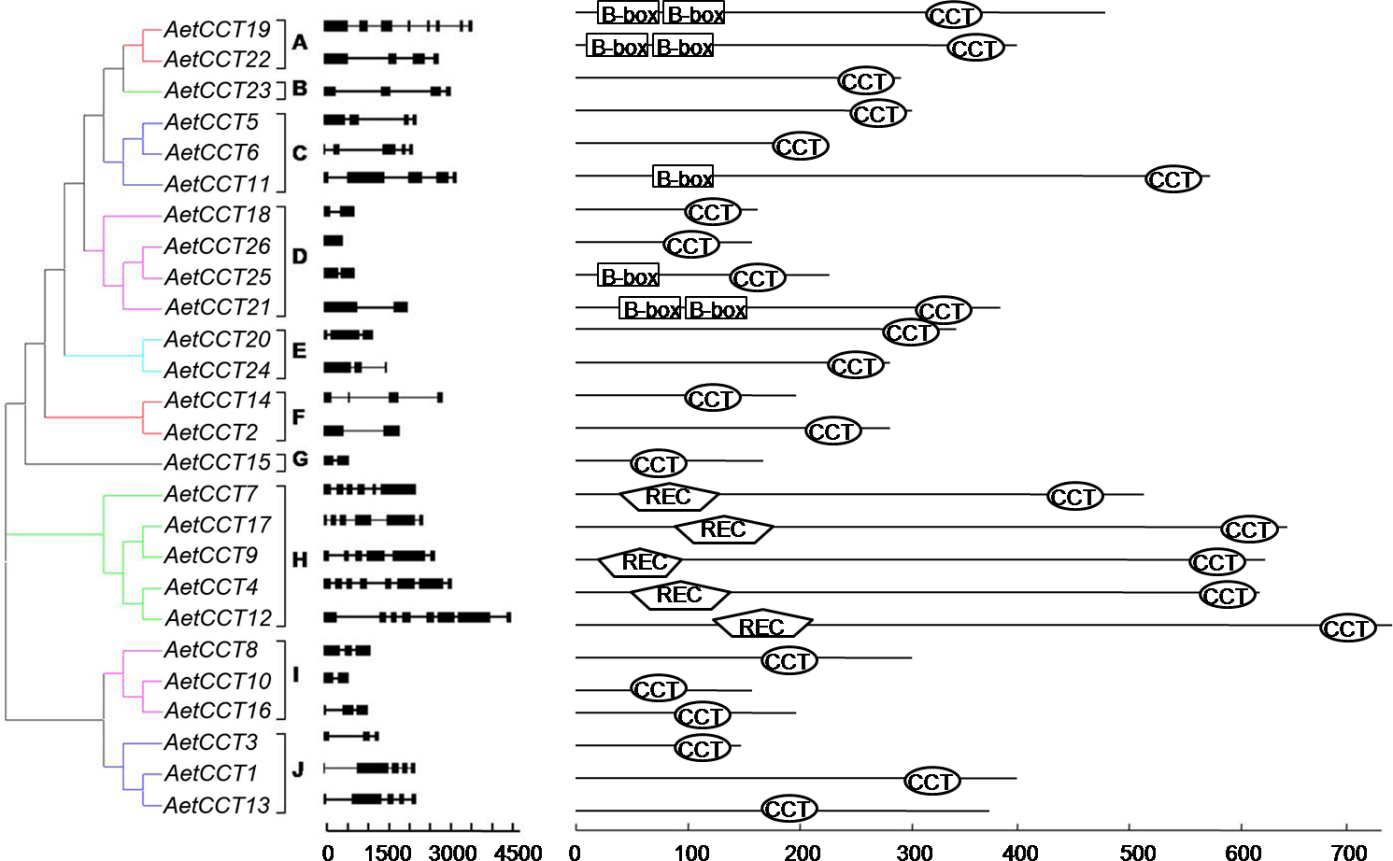
Phylogenetic relationship, gene structure and motifs of *AetCCT* genes. (a) The phylogenetic tree of *AetCCTs* constructed from a complete alignment of 26 *A*. *tauschi*. *CCT* genes using MEGA 6.0 by the N-J method with 1000 bootstrap replicates. Bootstrap scores are indicated on the nodes. (b) Exon/intron structures of *AetCCT* genes. Exons are represented by black boxes and introns by black lines. The sizes of exons and introns can be estimated using the scale below. (c) The conserved motifs of AetCCTs. Motifs were identified by SMART tool using the deduced amino-acid sequences of the AetCCTs.

*AetCCT* genes vary considerably in sequence length and gene structure (Fig 3). The longest gene sequence is *AetCCT12*, with a length of 4,521 bp, and the shortest is *AetCCT15*, just 603 bp. The genetic structure shows that there are 1, 6, 5, 4, 4, 3 and 3 members containing 0, 1, 2, 3, 4, 5, and 7 introns in *AetCCT* genes, respectively, and each CCT motif is encoded by the larger exons. Conserved motif analysis shows that the CCT domain is highly conserved among *AetCCT* genes. However, the configuration difference of introns and exons of different genes is large, and even gene introns and exons in the same group have obviously different configurations. The AetCCT motif distributions in the same group are similar, but the motif distribution between different groups has great differences. In addition, for some members of a group, the function and structure domain varies in species. For example, the rice homologous genes *Os08g42440* and *Os09g33550* of Group B contain a B-box structure domain, while this domain is missing in the homologous gene *AetCCT23*. These results suggest that *AetCCT* genes in the same group may have similar functions, and the specific motif of different members among families and within a family is likely to be the important reason for the various functional differences among different subfamily genes.

### Evolutionary rate analysis of *AetCCTs*

In order to further understand the evolutionary differences among different groups of *AetCCTs*, the evolutionary rate of each group is analyzed by PAML software. It can be seen in Table 2 that *AetCCT* genes have an obviously different evolutionary rate for different branches in the process of species formation, among which groups B, D, E and F have low evolutionary rates. While groups A, C, H and I achieve rapid evolution, the ω value of each branch is 0.471, 0.246, 0.71194 and 0.35719, respectively, suggesting that rapid evolution is related to the wide adaptability of species. In Fig 2, groups A, C, H and I include important flowering regulating genes related to adaptability known in Arabidopsis and rice. For example, group H in PRR subtribe has become an important factor in the photoperiodic response of Gramineous species in the process of speciation, which is crucial to the wide adaptability of species. *Os01g61900* in group I is related to the adaptability in rice, and the overexpression of *Os01g61900* can delay flowering for 14 days and 25 days under short-day and long-day conditions, respectively [19]. The gene delays rice heading by inhibiting the expression of *Ehd1*, *Hd3a* and *RFT1*, while there is no definite report on the function of homologous gene *AT5G41380* in Arabidopsis. Therefore, wide adaptability is likely related to the sustaining evolution of *AetCCT* family members of the groups, and the genetic alterations in cycle sensitivity allow *A*. *tauschii* to adapt to different environments.

**Table 2:** Evolutionary rates of different subgroups of 26 *AetCCTs*.

Model	-lnL	Estimated parameters	LRT statistic
One-ratio	-4541.21	ω0 = 0.20	
Nine-ratio	-4531.14	ω0 = 0.170(ωJ = ω0),**ωA = 0.471**,ωB = 0.007,**ωC = 0.246**, ωD = 0.00852,ωE = 0.00814,ωF = 0.00766,**ωH = 0.71194**, **ωI = 0.35719**,ωJ = 0.09898	20.14*

* P < 0.05. The statistical values of LRT were calculated from twice the log-likelihood difference between the two models.

### *AetCCT* positive selection test

In order to adapt to a new environment, differentiated species often needs to change the structure and function of specific proteins in order to meet new needs, so the positive selection of beneficial mutations in genes are likely occurred and passed on stably. Group A and B have a closer evolutionary proximity and evolve from the same ancestor, while group A (ωA = 0.471) and group B (ωB = 0.007) have different evolutionary rates after divergence. In order to detect the presence of positive selection in group A, the positive selection sites were detected in PAML using the Branch-site model. As shown in Table 3, 42.1% of the sites in group A had positive selection (ω = 76.579), among which four sites had a posteriori probability of more than 0.95, promoting divergence of *CCT* genes. *AT3G07650* and *AT2G33500* in group A are the circadian clock genes for long-day regulation [25, 26]; as the homologous gene of *AT3G07650*, *OsDTH2* (*Os02g49230*) is involved in regulating flowering in long-day conditions [9]. *CCT* genes of group A experienced the positive selection, may be due to the acquirement of new function in these genes after divergence, or relaxed purifying selection caused by losing the original function.

**Table 3:** Identification of positive selection sites in group A.

Model	-lnL	Estimated parameters	LRT statistic	Positive Sites
M1a (neutral)	-5751.08	p0 = 0.435 p1 = 0.201(p2+p3 = 0.364) ω0 = 0.181, ω1 = ω2 = 1		not allowed
M2a (selection)	-5749.25	p0 = 0.399 p1 = 0.180(p2+p3 = 0.421) ω0 = 0.184, ω2 = 76.579	3.66*	43I, 44W,74S,**100S**

* P < 0.05. The positive selection sites which P>0.95 are listed, and the bold site indicates P>0.95.

### Expression profile analysis of *CCT* genes in *A*. *tauschii* tissues

**Fig 4:**
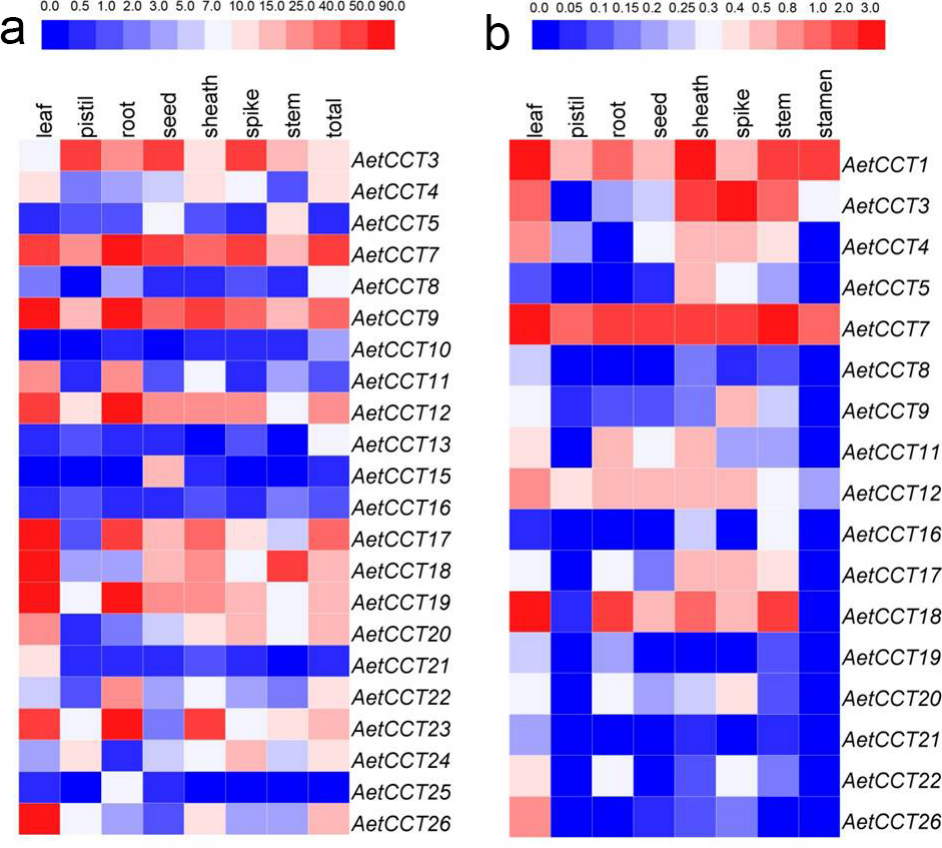
Heatmap representation for expression patterns of *AetCCT* genes across different tissues. (a) The expression profile data of *AetCCT* genes in leaf, pistil, root, seed, sheath, spike and stem were obtain from AGDB database. (b) Expression levels of *AetCCT* genes in 8 organs measured by qRT-PCR. The heatmap was generated using HemI (version 1.0.3.3). Higher and lower levels of transcript accumulation are indicated by red and blue, respectively, and the median level is indicated by white.

With the rapid development of sequencing technology, the public database contains a large amount of transcriptome and gene expression datasets of different growth periods and tissues, laying a solid foundation for the accurate prediction and analysis of the expression of *AetCCTs*. The analysis was made of the expression data of 22 *AetCCT* genes in 9 different tissues and organs obtained from AGDB database. It can be seen in Fig 4A that the *AetCCT* genes represent two different expression patterns. One is the constitutive-expression that genes have a relatively consistent expression level in all tissues and growth periods, such as *AetCCT3*, *4*, *7*, *9*, *12*, *17*, *18*, *22* and *26*. The other is the tissue-specific expression, by which *AetCCT15*, *AetCCT21* and *AetCCT25* have preferential accumulation of transcripts in seeds, leaves and roots, respectively. This shows that the members of the family have a certain diversity in both function and mode of action. In addition, the expression patterns of genes with similar structures are not always the same; different members may participate in different growth and development processes of *A*. *tauschii*, suggesting that different members participate in different metabolic pathways or different nodes on the metabolic pathways in the regulation of the flowering process of *A*. *tauschii*.

To further confirm the results of public expression datasets of *AetCCT* genes, qRT-PCR was performed for 17 *AetCCT* genes at 8 tissues of the *A*. *tauschii* (Fig 4B). The expression patterns of most of the *AetCCT* genes in the qRT-PCR analysis were consistent with transcriptome analysis, except the *AetCCT9* and *AetCCT19* showed higher expression in all tissues measured by transcriptome datasets. This may be explained by the sequencing bias occurred in the transcriptomic analysis.

### Response pattern of *AetCCT* genes to different hormones

**Fig 5:**
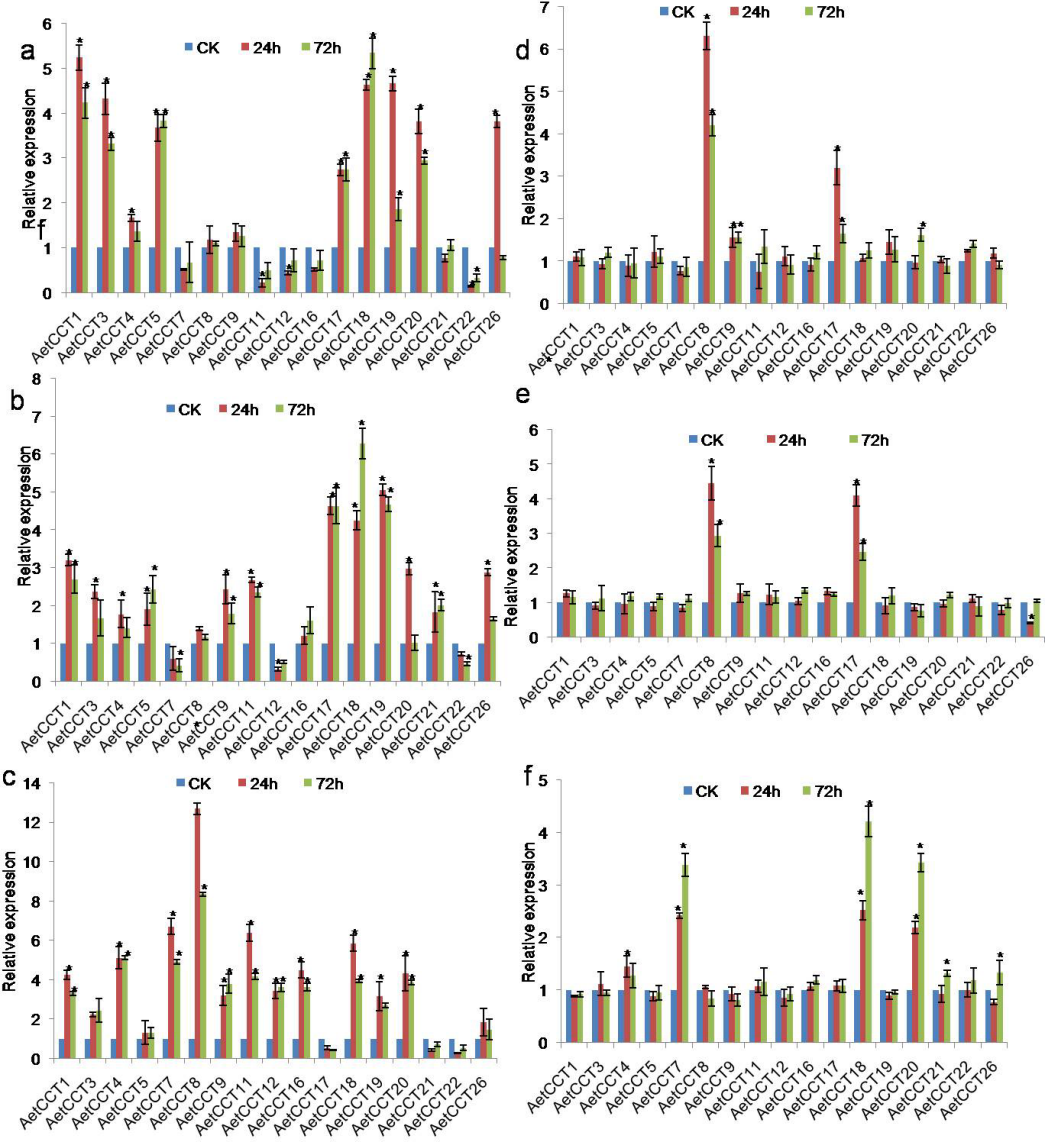
Expression analysis of *AetCCT* genes under treatments of different hormones. Plants at the booting stage were treated with 2 mM IAA **(a)**, 5 mM NAA **(b)**, 1 mM GA **(c)**, 1 Mm MeJA **(d)**, 15 Mm SA **(e)**, 1 mM BL **(f)**. The significant differences between data were calculated using Student’s *t* test, and indicated with an asterisks (*), *P*<0.05.

The variety of growth and development processes of plants are closely associated with hormones. As such, studying the expression changes of the *AetCCT* gene after being treated with different hormones can help to understand the function and mode of action of AetCCT members. A total of 17 *AetCCT* gene expression results were detected after being treated with IAA, NAA, BL, GA4, MeJA and SA (Fig 5), the 9 remaining gene expressions being too low to calculate. The gene expressions of *AetCCT* family members showed different responses to IAA, NAA, BL and GA4. *AetCCT17*, *21* and *22* were inhibited by gibberellin, while the rest experienced an enhancement effect in which the expression increased. Among them, the expressions of *AetCCT8* and *11* were highest at the 24 h after treatment, 12.7 times and 6.4 times higher than that of the control. Different members of *AetCCT* have a basically consistent response trend to IAA and NAA, two hormones that can induce the up-regulation expression of *AetCCT1*, *3*, *18*, *19* and *20*, while *AetCCT7*, *12*, and *22* showed inhibited expressions. Only a few members showed a response to BL, for instance, the expressions of *AetCCT7*, *18* and *20* increased. Most *AetCCT* members were not sensitive to MeJA and SA, and only a few members responded after treatment. Among them, the expressions of *AetCCT8* and *17* were high in the presence of MeJA and SA; while SA inhibited the expression of *AetCCT26*. Except for BL, *AetCCT17* had an obvious response to other 5 hormones. GA4 inhibited its expression, while the other four hormones enhanced it. *AetCCT4*, *7*, *18*, *19* and *20* showed a response to 4 hormones, and the different genes had a different response mechanism to the hormones. The overall response trend of each member to hormones was consistent in 24 h and 72 h after treatment. The expression level of most genes decreased in 72 h after experimental treatment. The expression of most genes 72 h after experimental treatment was lower than that of the 24 h group, but *AetCCT18* showed a higher expression in 72 h under the influence of IAA, NAA and BL. It can be seen that the function of the *CCT* family gene has a great differentiation and the involved hormone regulatory network is more diversified, which not only participates in the regulation of the flowering pathway, but also affects plant production, reproduction, adaptability and many other related traits.

### Expression responses of *AetCCT* genes to abiotic stress

**Fig 6:**
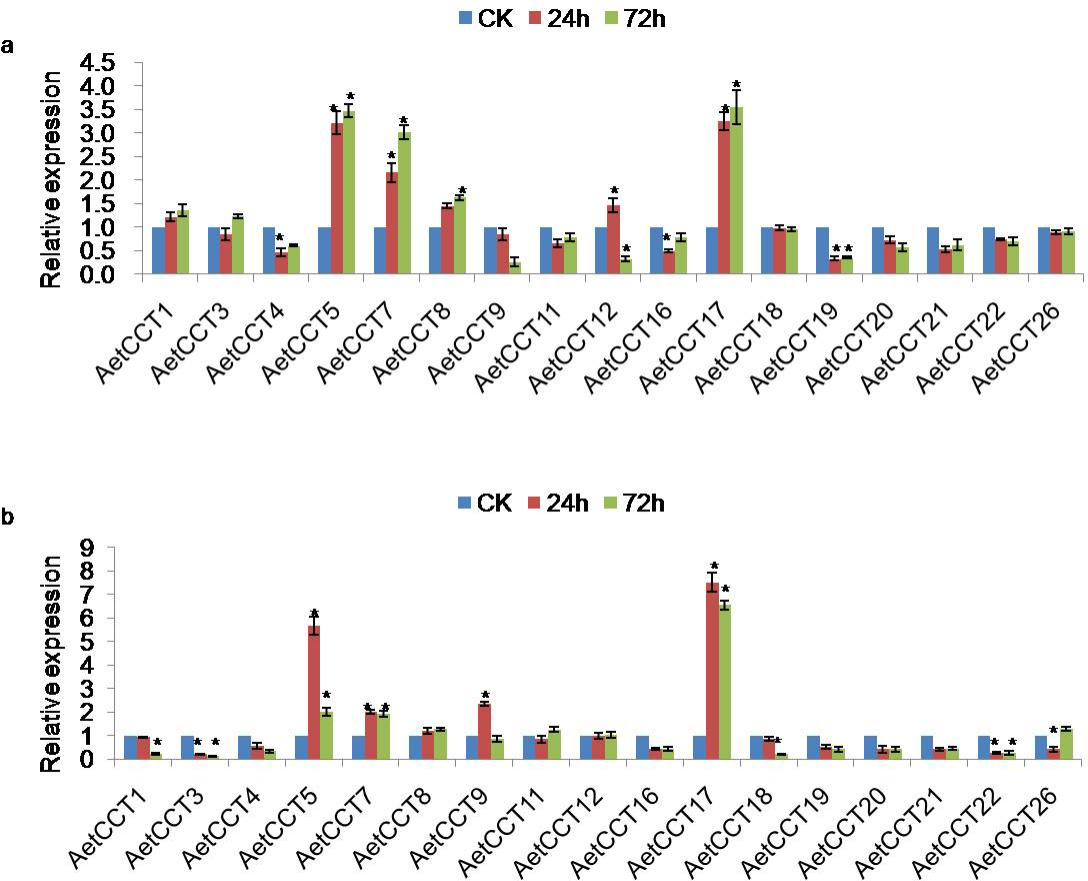
Expression of *AetCCT* genes in response to drought and salt treatment. **(a)** Changes in expression levels of 17 *AetCCT* genes at 24h and 72h after treatment with 15% PEG 6000. **(b)** Changes in expression levels of 17 *AetCCT* genes at 24h and 72h after treatment with 250 mM NaCl. The significant differences between data were calculated using Student’s *t* test, and indicated with an asterisks (*), *P*<0.05.

To investigate the roles of *AetCCT* genes in response to abiotic stresses, the expression profiles of *AetCCT* genes under drought and salt stresses were analyzed (Fig 6). Most *AetCCT* genes were regulated similarly in response to two abiotic stresses. *AetCCT5*, *7*, *8* and *17* were induced by both PEG and NaCl treatment, while *AetCCT4*, *16*, *19*, *20*, *21* and *22* showed down-regulation during 72h after the two treatments. Some genes were specifically induced or repressed when subjected to stress. For instance, *AetCCT1* was induced by PEG but repressed by NaCl, whereas *AetCCT9* was induced by NaCl but repressed by PEG. The qRT-PCR expression profiles exhibited different expression patterns for these *AetCCT* genes under specific treatments, thus providing a useful resource for further functional analyses.

### Expression patterns of *AetCCT* genes under the light cycle

**Fig 7:**
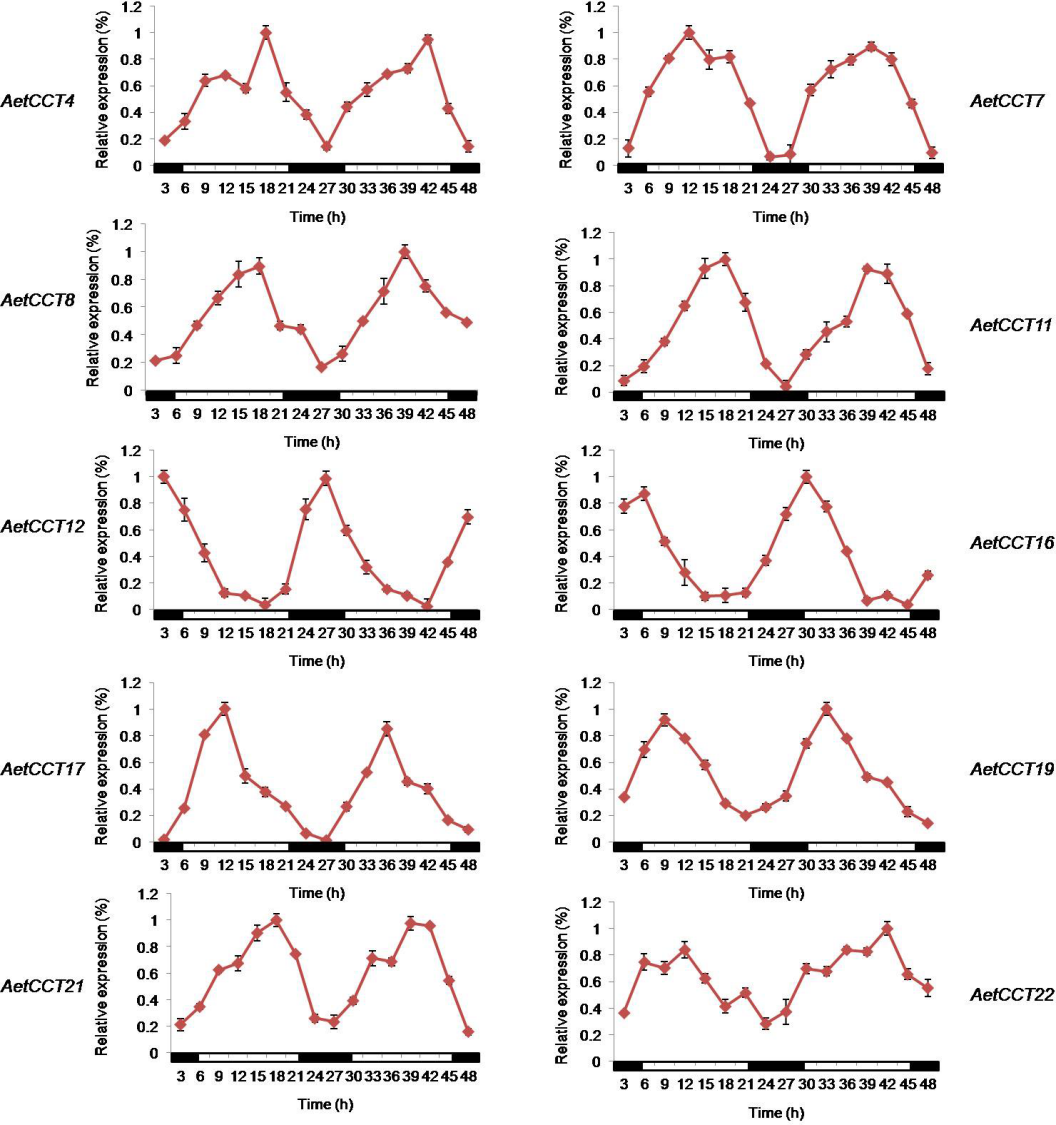
Expression patterns of *CCT* domain genes during 48h. The white and black bars represent the light and dark periods, respectively. Mean values ± SD were obtained from three technical repeats and two biological repeats.

*CCT* family genes participate in the regulation of plant flowering time, and the related genes can regulate the light cycle through the circadian clock effect of *CCT* family genes. Therefore, the circadian expressions of 17 *AetCCT* genes were studied (Fig 7). The results of the test show that the expressions of *AetCCT4*, *7*, *8*, *11*, *12*, *16*, *17*, *19*, *21* and *22* have an obvious circadian clock effect with a 24 h rhythmic expression, indicating that these members can feel the signal of light cycle changes, then regulate their own growth and development processes. Among them, *AetCCT4*, *11*, *17*, *19* and *21* were expressed under light conditions, and the expression increased with the illumination time and gradually decreased under darkness conditions. *AetCCT7*, *8* and *16* were mainly expressed during the day, and achieved the peak in a moment. *AetCCT12* and *AetCCT 22* were mainly expressed under dark conditions, and achieved the highest expression before dawn; its expression was inhibited in light conditions. Among members with no circadian expression, different members also showed different expression (S1 Fig). For example, the difference of expression strength of *AetCCT20* at each time point within the 24 h cycle was small, while the difference of expression strength of *AetCCT1*, *7*, and *9* at each time point was large. It is worth noting that 9 of the 10 members with a circadian clock effect are in the branch of rapid evolution in *A*. *tauschii*; *AetCCT19* and *22* belong to group A; *AetCCT4*, *7*, *12* and *17* belong to group H; and *AetCCT8* and *16* belong to group I, further indicating that groups A, C, H and I may play a role in the formation process of the adaptability of *A*. *tauschii*.

## Discussion

### The CCT gene is an important gene for the life metabolism of *A*. *tauschii*

Flowering is an important life process of plants which has a great influence on the reproductive cycle and yield. *CCT* genes exist widely in gymnosperms and angiosperms, most members of which exercise an important role in the control process of flowering. The long-term evolution and selection of different species has resulted in the *CCT* gene function having great differentiation between species, and more diversified gene regulation methods, which not only participate in the regulation of the flowering pathway, but also affect plant production, reproduction, adaptability and many other related traits. For example, *Ghd7* inhibits rice flowering under a long photoperiod, and the height and yield per plant of mutants is increased significantly [5]; the corn homologous gene *Ghd7* controls the sensitivity of the corn to light and influences the spread of the corn after domestication by the methylation degree of the CACTA transposon cis element [27]. Griffiths *et al*. made the first comprehensive analysis of the *CO* gene isolated from Arabidopsis, rice and barley. The *CO* was classified according to its gene structure and the homology of its amino acid [28]. Cockram *et al*. recounted and reclassified the *CCT* family genes through the integration of the latest sequencing information of Arabidopsis, rice, sorghum and barley [29]. In this research, 26 *AetCCT* genes were identified and divided into 10 subgroups, respectively belonging to different subfamilies. The genetic relationship of *CCT* genes between *A*. *tauschii* and wheat is the most closed, followed by *T*. *urartu*, rice and Arabidopsis, which conforms to the general rules of plant species formation for Gramineae species. *AetCCT15* has species specificity, suggesting that it may have new functions, and in groups A, D, F, I, J and H contain the functionally characterized *CCT* genes from other species, which can provide a reference for the functional research of the *AetCCT* genes. In addition, half of the AetCCT members are in the region near the centromere on the chromosome. The recombination frequency of genes in this region tends to be lower in the evolutionary process, suggesting that these AetCCT members have strong conservation in terms of function and structure. Common wheat is allohexaploid (AABBDD) and its genome is huge, mostly consisting of repetitive sequences, which has led to the circumstance in which research progress in wheat is significantly lagging behind that of rice, corn and other crops. It restricts the foundation research process of wheat yield and quality improvement. As a donor of the D genome of wheat, *A*. *tauschii* gives wheat better adaptability and quality traits, and the completion of genome sequencing has laid a good foundation for research into the adaptability and quality of wheat. As such, the analysis of information on the chromosomal location, sequence signature, gene structure, evolution and so on of the AetCCT family members helps us to understand the formation mechanism of the wide adaptability of *A*. *tauschii* and wheat.

### AetCCTs have functional diversity

It has been found that about 60% of the genes in plants are regulated by the circadian clock. Research into the circadian clock genes of Arabidopsis is more in-depth, and some key genes such as *TOC1*, *TEJ* and *FIONA1* have been successively identified [30–32]. Genes such as *EARLY FLOWERING3*, *EARLY FLOWERING4* and *GIGANTEA* have been found in succession through screening mutants [33, 34], so in-depth study of the effects of the circadian clock has important significance for understanding the mechanism of plant development. According to our analysis, there is significant ' circadian clock effect' in the gene expression of 10 gene members, presenting a rhythmic expression of 24 h. In Arabidopsis, the *PRR5*, *7* and *9* of the CCT family are sequentially expressed in turn during the day, *PRR9* works in the morning, *PRR7* is in an active state from morning to midnight, and *PRR5* keeps functioning from noon to midnight [35]. In the present study, we found that the expression levels of *AetCCT7*, *8* and *16* were highest when exposed to light for 9h, the expression levels of *AetCCT4*, *11*, *19* and *21* were highest before dark, and *AetCCT4* and *11* functioned at night, which fully explains how these *AetCCT* genes commonly regulate the life activities in the operating mechanism of the circadian clock in *A*. *tauschii*. Light cycle gene function is sometimes affected by posttranslational modifications; for example, phosphorylation modification after translation is crucial for the nuclear localization and signal recognition of *CCA1* and *ZTL* [36, 37]. Therefore, although the circadian expression rhythms of *AetCCT1*, *3*, *5*, *7*, *9*, *18*, *20* and *26* were not found, it is possible for them to function as clock proteins through subsequent modification.

On this basis, this study further analyzed the responses of AetCCT to six exogenous hormones, and further explored the mechanisms of different members participating in the regulation. The results show that all members of AetCCT are very sensitive to GA4, GA4 can inhibit the expression of *AetCCT17* and *AetCCT22*, the expressions of remaining members are increased in the presence of GA4, and *AetCCT8* is the most sensitive, especially after 24h treatment, enhancing 12 times compared to the control. It is speculated that most *AetCCT* genes are involved in the light reaction metabolism of *A*. *tauschii* via the gibberellin signaling pathway. In addition, as IAA and NAA are involved in almost all growth and metabolic processes of plants [38, 39], the members of the *AetCCT* gene involved in the IAA and NAA metabolism pathways are more numerous, and each member has an obvious synergistic effect for the two hormones; for example, both IAA and NAA have promotion effects on the expression of *AetCCT1*, *AetCCT19* and *AetCCT20*, while inhibiting the gene expressions of *AetCCT7*, *AetCCT12* and *AetCCT22*. In addition, members sensitive to IAA and NAA show constitutive expression according to the results of tissue-specific expression, and it is speculated that AetCCT is involved in the growth and metabolism of *A*. *tauschii* mainly through the network of these two kinds of hormone. The overall trend of each member's response to hormones after hormone application for 72h is consistent with that after 24h, but the expressions of most genes were decreased, which may be related to self-metabolism or the degradation of exogenous hormones in plants. The hormone response results verify that the function of *CCT* family genes has a high conservation in the evolution of species.

Plant responses to abiotic stress are mainly mediated by phytohormones, for instance, MeJA and SA play a role in responses to abiotic stresses and pathogens. Grundy et al. revealed circadian regulation by PRR5, PRR7 and TOC1 affects the stress-responsive hormonal pathways in Arabidopsis [40]. Circadian clock controls expression of a large fraction of abiotic stress-responsive genes, as well as biosynthesis and signaling downstream of stress response hormones. In this study, *AetCCT8* and *AetCCT17* with rhythmic expression pattern were significantly induced by drought and salt stresses. Both of them were seen to have been increased in the presence of MeJA and SA, which may reveal an effect of *CCT* genes on regulation of the stress-responsive hormone signaling pathways in *A*. *tauschii*.

### Rapid evolution of the *CCT* gene family promotes the adaptability of *A*. *tauschii*

From researching into Archaea, Groussin *et al*. found that the rapid evolution of gene families can improve species adaptability in the evolution process; the evolutionary rates of some genes gradually increased and their accelerated evolution promoted the adaptations of Archaea during the gradual reduction of the earth's environment temperature [41]. After the phylogenetic analysis of 11S globulin protein genes of dicots and monocots, Li *et al*. found that significant positive selection could be detected in the evolutionary processes of cucumber, poplar, rice, Arabidopsis and other dicots, which was not detected in the evolutionary process of monocots [42]. Relevant research into the presence of the universality of this regularity in plants is not so plentiful. *A*. *tauschii* is mainly distributed between 30°-45° north latitude, and West Asia, the Middle East, southeast Europe, northern Africa and the Mediterranean are major global normal regions of *A*. *tauschii*. The latest studies suggest that *A*. *tauschii* shows a wide adaptation range in its morphology and ecology, and the suitable scope continues to expand [43]. The cause of this phenomenon may be that natural selection improved its adaptive capacity to different regions. The CCT gene is the main regulatory factor of a plant to adapt to the environment. The related members are bound to suffer selection, and the differential selection often provides the molecular basis for the evolution and adaptation of species. The model analysis in this study found that groups A, C, H and I showed the phenomenon of rapid evolution, indicating that the branch members may be related to the adaptation ability of *A*. *tauschii*. Because *A*. *tauschii* had to adapt to the photoperiods of different regions, the *CCT* gene needs more variations to adapt to environmental changes, and positive selection has retained more favorable variations, so that these genes have a high evolution speed. In addition, the weed type *A*. *tauschii* in the Yellow River Basin of China must have originated in Iran or adjacent areas, and recent studies have shown that the Shanxi and Henan weed type of *A*. *tauschii* in different ecological regions has undergone obvious genetic differentiation [44], suggesting that natural selection can speed up species evolution and improve the adaptability of the population.

In addition, studies have reported that the evolution rate is strongly associated with the metabolic rate. Having studied fish and mammals, Gillooly *et al*. found that if the living environment temperature was increased for 10 degrees, the genetic evolution rate of fish and mammals would be increased by 300%; that is to say, the related genes evolve faster as the metabolic rate grows faster [45]. Having researched the ecological adaptability of *A*. *tauschii* in different areas, Fang *et al*. found that along with global climate change and increased greenhouse gas emissions, *A*. *tauschii* showed a successive expansion trend in the global normal region and a reduction trend in the low normal region and comfortable normal region in the emissions scenarios of A2a and B2a, while the metabolism of *A*. *tauschii* sped up in some areas, presenting a trend of increased height which was consistent with research results concerning the evolution rate and metabolic rate of animals [43]. The phylogenetic analysis results of this study show that some branches of AetCCT show a positive selection effect, rapid evolution improves the adaptability of *A*. *tauschii*, and the AetCCT family is the important regulatory factor of the light reaction process, growth and metabolism in *A*. *tauschii* with functional diversity. Therefore, the results of this paper not only provide useful information for wheat evolution studies, but also provide a theoretical basis for the comprehensive control and ecological characteristics of the weed type *A*. *tauschii*.

## Supporting information

S1 Fig Expression patterns of Aet*CCTs* with no circadian clock effect during 48h. (TIF)

S1 Table List of qRT-PCR primers used in this study. (XLSX)

S2 Table Orthologue *CCT* genes in rice and Arabidopsis. (XLSX)
