# Supplementary figures and images for: Characterization of the CCT family and analysis of gene expression in Aegilops tauschii
Source: PLoS One. 2017 Dec 8;12(12):e0189333. doi: 10.1371/journal.pone.0189333 (PMC5722339; doi:10.1371/journal.pone.0189333)

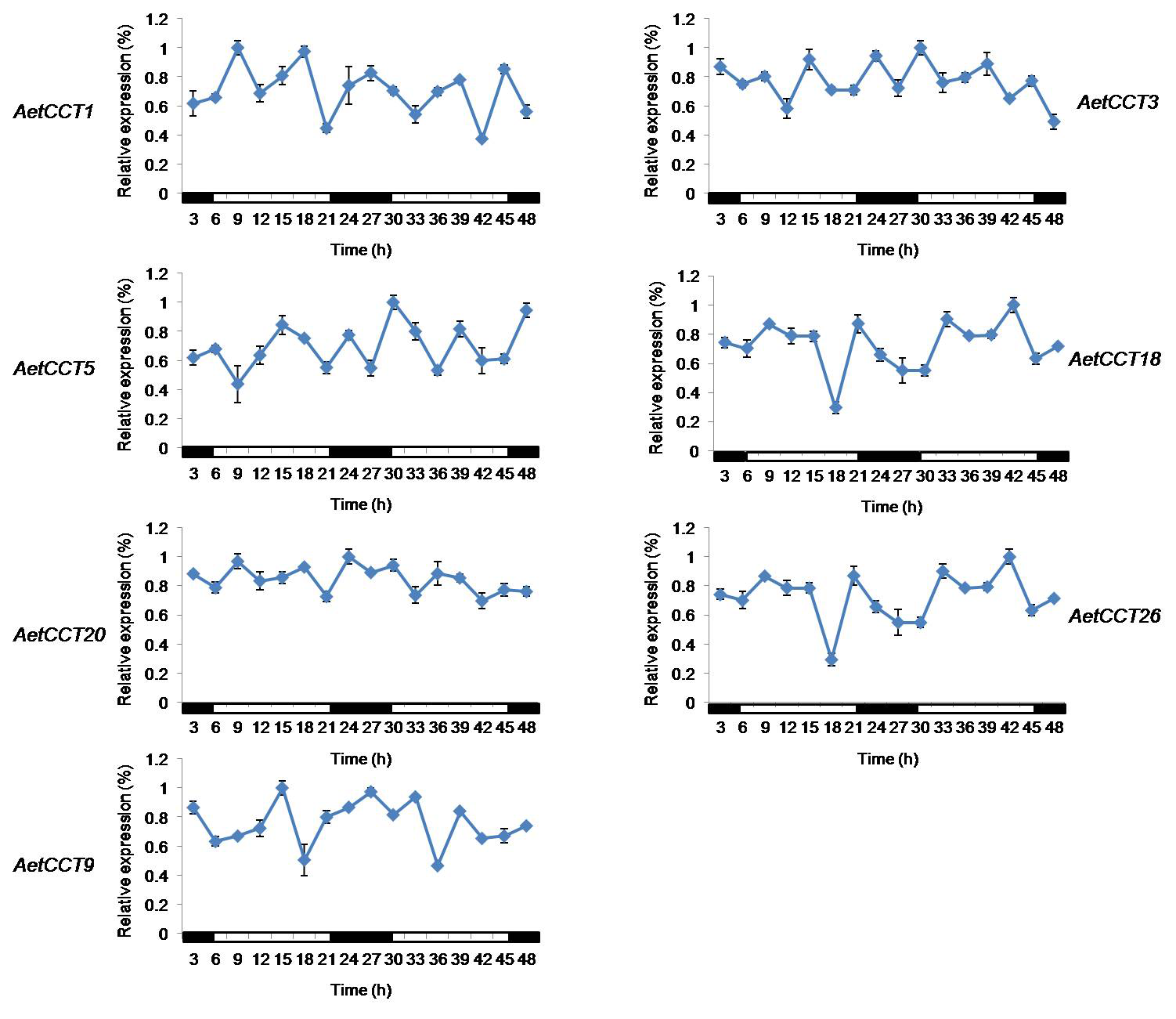

Supplement: S1 Fig — (TIF) [file pone.0189333.s001.tif]
